# Small bowel perforation caused by thermal injury during colonic polypectomy: A case report and literature review

**DOI:** 10.1097/MD.0000000000029681

**Published:** 2022-08-05

**Authors:** Cong Yuan, Kui Xu, Guo-Dong Yang, Chun-Hui Xi, Xue-Mei Lin

**Affiliations:** a Department of Gastroenterology, Affiliated Hospital of North Sichuan Medical College, Nanchong, Sichuan, China; b Department of Gastroenterology, People’s Hospital of Yuxi City, Yuxi, Yunnan, China; c Department of Pathology, Basic Medical College of North Sichuan Medical College, Nanchong Sichuan, China; d Department of Pathology, Affiliated Hospital of North Sichuan Medical College, Nanchong, Sichuan, China.

**Keywords:** colonoscopy, polypectomy, small bowel perforation, thermal injury

## Abstract

**Rationale:**

Iatrogenic gastrointestinal perforation is a known uncommon complication of colonoscopy. The perforation usually occurs in the colon itself. Rarely, colonoscopic procedures can also cause the perforations of the small intestine.

**Patient concerns and diagnoses:**

We describe the case of a 70-year-old man who experienced abdominal pain several hours after electrical polypectomy in the transverse colon. Urgent abdominal computed tomography scans showed a few bubbles on the frontal surface around the liver and a little extraluminal free air in the upper abdomen. Urgent exploratory laparotomy revealed a round perforation with a diameter of approximately 5 mm in the ileum 80 cm proximal to the ileocecal valve, accompanied by the outflow of intestinal contents. A small bowel perforation by thermal injury was diagnosed during colonic polypectomy.

**Interventions and outcomes:**

The ileal perforation was repaired primarily after debridement of the perforation site and abdominal cavity. The patient recovered well after surgery. Histopathological examination of the perforation site demonstrated inflammatory necrosis and infiltration of inflammatory cells.

**Lessons:**

Small bowel perforation should be considered after colonoscopic procedures although the incidence is exceedingly rare. Urgent exploratory laparotomy is warranted when a visceral perforation is identified after colonoscopy.

## 1. Introduction

Colonic perforation is a known complication following colonoscopy, occurring at the frequency of 0.012% to 0.084%.^[1,2]^ Perforation of the small intestine after colonoscopy is extremely less common. Herein we present a case of small bowel perforation by thermal injury after electrical polypectomy in the transverse colon. A literature review of small bowel perforation after colonoscopy is included in the PubMed database from 1976 to the present. This rare complication of colonoscopy is summarized to alert the endoscopists to the possibility of perforations occurring in areas remote from the colon.

## 2. Case report

**Figure 1.**
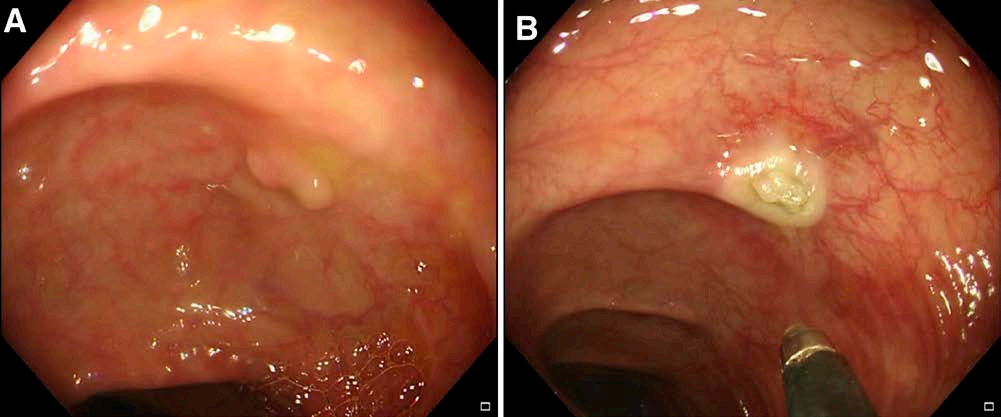
Colonoscopy showed a sessile polyp with about 5 mm in diameter in the transverse colon (A), which was coagulated by radiofrequency therapy (B).

A 70-year-old man underwent colonoscopy for follow-up of colonic polyps. He had no history of abdominal surgeries except for prior colonoscopic polypectomy. After routine preoperative examinations, colonoscopy (CF-H290I; Olympus Optical Co. Ltd, Tokyo, Japan) screening was carried out under general anesthesia. Following the whole colorectal examination, only a sessile 5-mm polyp in diameter was found in the transverse colon (Fig. 1A). Then, according to the operation manual of radiofrequency therapeutic instrument (XVC-II radiofrequency therapeutic instrument, Xi’an Gaotong Technology Development Co. Ltd, Xi’an, China), radiofrequency coagulation was performed by a licensed physician who has been consecutively engaged in the radiofrequency polypectomy for >20 years (Fig. 1B).

**Figure 2.**
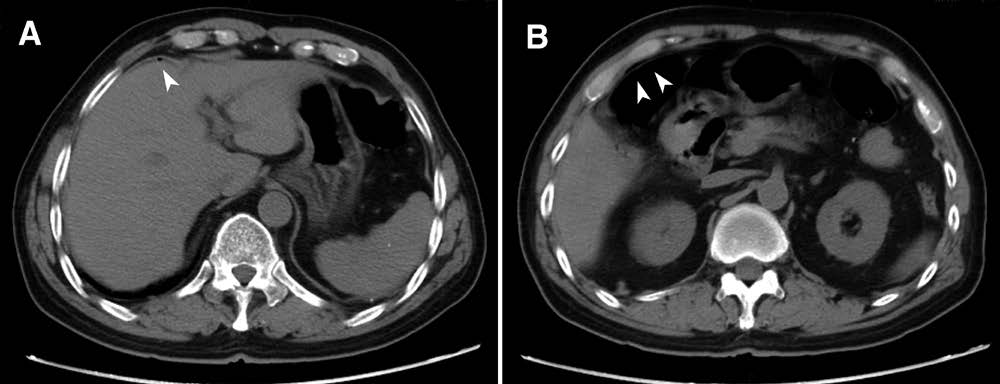
Urgent abdominal computed tomography scans showed a few bubbles on the frontal surface around the liver (A, arrowhead) and a little extraluminal free air in the upper abdomen (B, arrowhead). No peritoneal effusion was found.

Five hours later, the patient complained of slight abdominal distention and periumbilical pain. On examination, the patient was afebrile with normal vital signs. Abdominal palpation indicated mild local peritonitis confined to the periumbilical region. Leucocyte count was normal. Abdominal plain film showed no free gas under the diaphragm. An abdominal computed tomography scans revealed a few bubbles on the frontal surface around the liver and a little extraluminal free air in the upper abdomen, and no peritoneal effusion was found (Fig. 2). Based on the fact that visceral perforation occurred after colonic polypectomy, it was speculated that the perforation position was most likely to be located at the site of colonic polypectomy. Given the early stage of abdominal perforation and slight abdominal manifestations, an urgent colonoscopic procedure was implemented with as little carbon dioxide insufflation as possible. However, no visible perforations were found in the whole colon, except that the polyp resection site was covered with white exudate. The polypectomy site was still clipped with endoclips.

**Figure 3.**
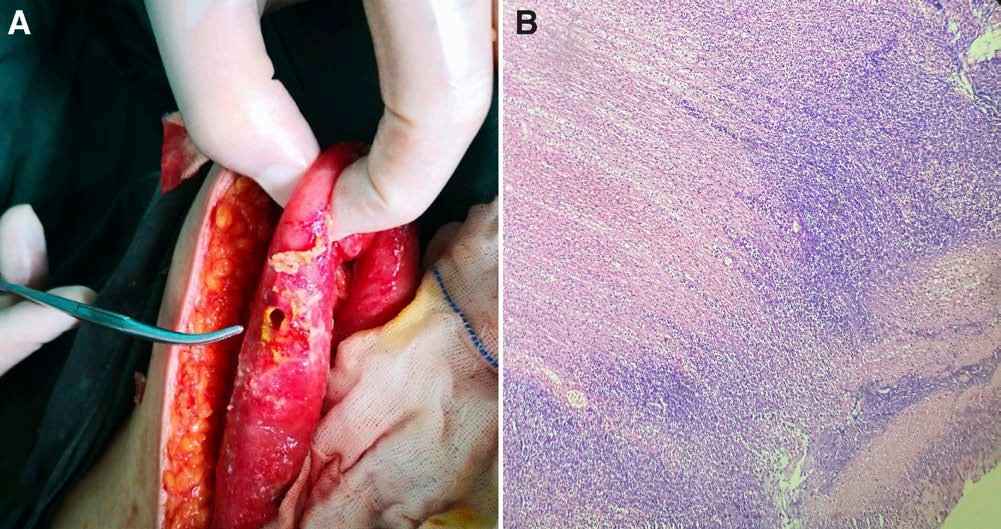
Urgent exploratory laparotomy displayed a round-shaped perforation with a diameter of about 5 mm in the ileum 80 cm proximal to the ileocecal valve, accompanied by the outflow of intestinal contents (A). Pathological examination demonstrated that there were many inflammatory debris and inflammatory exudates near the perforation site (B). (HE, ×40). HE = hematoxylin–eosin staining.

After consulting the condition in time, the patient asked to be temporarily managed conservatively. In the next few hours, the patient’s abdominal signs continued to worsen. His white blood cell count was elevated to 12,000/mm^3^ (normal range, 4000–9500/mm^3^). Emergency exploratory laparotomy was conducted. At laparotomy, liquid enteric contents were found in the peritoneal cavity. A cautery burn was uncovered at the polypectomy site of the transverse colon about 30 cm proximal to splenic flexure of colon, without evidence of clinically visible colonic perforation. A round-shaped perforation with a diameter of about 5 mm was found in the ileum 80 cm proximal to the ileocecal valve, accompanied by the outflow of intestinal contents (Fig. 3A). No additional perforations were discovered throughout the alimentary tract. The ileal perforation was repaired primarily after debridement of the perforation site and abdominal cavity, and the transverse colon injury was reinforced. The patient experienced well after surgery. Histopathological findings of the small intestinal perforation site demonstrated inflammatory necrosis and infiltration of inflammatory cells (Fig. 3B).

## 3. Discussion

Colonoscopy plays a vital role in the diagnosis and treatment of various colonic diseases, and it is generally approved to be safe. Colonic perforation is a known complication following colonoscopy, occurring at a very low incidence rate.^[1,2]^ Typically, iatrogenic gastrointestinal perforations after colonoscopy primarily occur in the colon itself.^[1,2]^ It is amazing that, in addition to colonic perforation, this procedure can also cause small bowel perforation. So far, the accumulative cases of small bowel perforation after colonoscopy are extremely rare. A literature review of cases from 1976 to the present was conducted in the PubMed database. Only 9 case reports were noted in the literature (Table 1).^[9]^ This rare complication of colonoscopy is summarized to alert the endoscopists to the possibility of perforations occurring in areas remote from the colon.

**Table 1:** Small bowel perforation cases after colonoscopy and the proposed mechanisms.

Year	Author	Patient characteristics					
		Sex	Age (yr)	Symptoms	Colonoscopy type	Perforation segments	Perforation sites
1976	Razzak et al^[3]^	F	69	Rectal pain, diarrhea and bloody mucus stool	Diagnostic	Distended loops of ileum	1
1994	Nijhawan et al^[4]^	M	60	Fever and maroon colored stools	Diagnostic	Inflammatory jejunum	3
1994	Nemeh et al^[5]^	F	84	Guaiac positive stool	Diagnostic	Ileum	2
1998	Chau et al^[6]^	M	61	Bloody diarrhea and abdominal pain	Diagnostic	Underlying ischemic ileum	1
2003	González et al^[7]^	F	62	Changes in intestinal habits	Diagnostic	Dilated loop of small bowel ileus	1
2008	Pasumarthy et al^[8]^	F	88	Follow-up of colonic polyps	Diagnostic	Jejunal diverticulum	1
2011	Tung et al^[9]^	M	60	Surveillance colonoscopy	Diagnostic	Jejunal diverticulum	1
1979	Erdman et al^[10]^	M	56	Multiple colorectal polyps	Therapeutic	Ileum	2
2007	Lambert et al^[11]^	F	67	20-mm polyp in the cecum	Therapeutic	Ileum	1
2022	Present case	M	70	5-mm polyp in transverse colon	Therapeutic	Ileum	1
		**Patient characteristics**					
**Year**	**Author**	**Size (mm)**	**Proposed mechanism**	**Electrocoagulated sites**	**Abdominal surgery history/adhesions**	**Prognosis**	
1976	Razzak et al^[3]^	18	Air insufflation	–	Hysterectomy and bilateral salpingo-oophorectomy/dense adhesions	Well	
1994	Nijhawan et al^[4]^	NA	Air insufflation	–	NA	NA	
1994	Nemeh et al^[5]^	50	Mechanical injuries (traction)	–	Right hemicolectomy for adenocarcinoma, cholecystectomy, hysterectomy with bilateral salpingo-oophorectomy/dense fixed pelvic adhesions	Well	
1998	Chau et al^[6]^	40	Air insufflation	–	No surgery	Well	
2003	González et al^[7]^	Small*	Air insufflation	–	Hysterectomy and appendectomy/peritoneal adhesions	NA	
2008	Pasumarthy et al^[8]^	NA	Air insufflation	–	NA	Well	
2011	Tung et al^[9]^	2	Air insufflation	–	Open right hemicolectomy for ascending colon cancer/extensive adhesions	Well	
1979	Erdman et al^[10]^	6–8	Thermal injury	Left transverse colon and rectum	NA	Well	
2007	Lambert et al^[11]^	NA	Thermal injury	Cecum	NA	Well	
2022	Present case	5	Thermal injury	Transverse colon	Peritoneal adhesions	Well	

The demographic data, colonoscopy type, perforation details, history of abdominal surgery, and proposed mechanisms of the cases are summarized in Table 1. There were 5 female and 5 male patients, with an average age of 67.7 years (mean ± standard deviation, 67.7 ± 10.6 years; range 56–88 years). Diagnostic colonoscopy was conducted in 7 cases and therapeutic colonoscopy in 3 cases. Seven patients underwent diagnostic colonoscopy because of numerous complaints, including abdominal pain, diarrhea, abnormal stool and habits, and postoperative surveillance for colonic polyps and carcinoma.^[3–9]^ The perforation segments were located in the ileum and jejunum. The size of perforation varied from several millimeters to 50 mm,^[5]^ and the number of perforations ranged between 1 and 3 sites in each patient. Among them, the perforations in 2 cases happened just over the jejunal diverticulum.^[8,9]^ The other 2 cases also had underlying lesions of the small bowel, such as intestinal inflammation and ischemia.^[4,6]^ In the next 2 cases,^[3,7]^ the perforations occurred at the dilated small bowel loop or ileus loop, and both cases had a history of abdominal surgery and intraperitoneal adhesions. In the last case of diagnostic colonoscopy.^[5]^ Two sites of ileal perforation were identified, one of which was measured nearly 50 mm in length. The patient had experienced several abdominal operations previously and dense intraperitoneal adhesions developed and fixed. In addition to the present case (Table 1), a total of 3 therapeutic colonoscopy patients underwent electrical resection for colorectal polyps.^[10,11]^ The perforation segments caused by electrical burn were all located in the ileum without underlying diseases, and the size of the perforation was confined to several millimeters. The number of perforations was 1 to 2 sites in each case. All ten cases underwent laparotomy intervention from several hours to 6 days^[6]^ after colonoscopy. Except for 2 cases whose prognosis was not described in the literature,^[4,7]^ 8 cases recovered well.

Iqbal et al^[2]^ had summarized that colonic perforations usually were caused by 3 mechanisms, including mechanical injuries (direct colonoscopic trauma or traction), local removal of tissue (e.g., polypectomy, endoscopic mucosal resection, endoscopic submucosal dissection, etc), and electrocautery. At present, the exact mechanism of small bowel perforation secondary to colonoscopic procedure remains obscure. Based on the limited case data currently available, 3 proposed mechanisms may be involved in the development of intestinal perforation: excessive air insufflation into the small intestine (namely, pneumatic injury), which may be accompanied by an incompetent ileocecal valve, thermal injury, and mechanical injury (Table 1).^[9]^ Six out of 7 patients with small bowel perforation after diagnostic colonoscopy were related to air insufflation.^[3,4,6–9]^ This mechanism was further strengthened by 2 cases, in which the colonoscope passed only 15 cm^[3]^ and 60 cm^[4]^ into the colorectum, and then perforations of the small intestine occurred a few hours later after the colonoscopy. The presence of underlying small bowel disease (e.g., diverticulum,^[8,9]^ ischemia,^[6]^ and inflammation^[4]^) might be a risk factor that made the small bowel vulnerable to perforate when the excessive air was insufflated into the small intestine through the incompetent ileocecal valve or removed ileocecum.^[9]^ Abdominal adhesions might be another predisposing factor. Adhesive bands might interfere with air escape during colonoscopy.^[6]^ This condition could lead to strangulation of the small bowel loop. A massive amount of air entering the small intestine would increase the intraluminal pressure, causing dilation of the intestinal loops, and even intestinal ileus.^[3,7]^ Further deterioration would give rise to small bowel perforation. Some authors suggest that the patients with these risk factors should benefit from the added safety and comfort by inflating carbon dioxide rather than room air.^[6]^ The second proposed mechanism was thermal injury during therapeutic colonoscopy.^[10,11]^ It was postulated that during colonic polypectomy, current transmission could occur from the colon wall to the adjacent small bowel, resulting in small bowel perforation.^[9]^ The round and small-size acute perforation in the ileum pointed directly to the electrical injuries rather than mechanical injuries.^[2]^ Consistent with the prior reported case of electric injuries,^[10]^ the size of the small intestinal perforation in the present case was also limited to a few millimeters. Safe endoscopic electrocoagulation requires that the electrode must have sufficient contact area with the surface skin to afford ready escape of electrons to avoid skin overheating. If this area is less than a certain critical size, skin burns may occur.^[10]^ Inspired by this phenomenon, the mechanism investigators speculated was that a readily conductive fluid-filled loop of the small bowel might be adjacent tightly to the electrocoagulated colon wall.^[10,11]^ A relatively small area in the ileal loop close to the colon wall increased the conductance. The current directly arrived at the ileum wall and caused cauterant perforation.^[10]^ The third mechanism was mechanical injuries. The patient had a history of multiple abdominal operations and extensive adhesions formed.^[5]^ The dense adhesions between the small bowel, colon, and abdominal wall created fixed points, which were torn when advancing the colonoscope and 2 perforation sites appeared. In such patients, gentle manipulation was crucial and should be abandoned when appropriate.

The present case was reexamined by urgent colonoscopy timely at an early stage after visceral perforation was identified. Unfortunately, the perforation did not be disclosed in the colon. In this situation, small bowel perforation should be considered. Based on all interventions reported so far, once small intestinal perforation occurred after colonoscopy, an urgent exploratory laparotomy was necessary rather than conservative treatment. Finally, proper use of electrocautery was necessary. Small (5–9 mm) colorectal polyps could be safely and effectively removed by cold snare polypectomy,^[12]^ which completely avoided the potential risk of electrical burns.

## 4. Conclusion

Overall, in patients with predisposing factors, such as small bowel diverticulum, inflammation, ischemia, or dense intraperitoneal adhesions, air inflation should be properly controlled during colonoscopy, and carbon dioxide insufflation may be recommended. The manipulation should be gentle and suspended when appropriate. The small bowel perforation should be borne in mind in the patient who experienced a colonoscopic procedure although the incidence is very rare. Urgent exploratory laparotomy is warranted when visceral perforation occurs after colonoscopy and no definite perforation by remedial emergency colonoscopy is found in the colon.

## Acknowledgments

We appreciated the patient from the People’s Hospital of Yuxi City for giving us the opportunity to share the case report with all of you.

## Author contributions

Conceptualization: Cong Yuan.

Data curation: Cong Yuan, Kui Xu, Chun-Hui Xi.

Formal analysis: Cong Yuan, Kui Xu, Chun-Hui Xi, Xue-Mei Lin.

Investigation: Cong Yuan, Kui Xu, Chun-Hui Xi.

Methodology: Cong Yuan, Kui Xu, Guo-Dong Yang.

Project administration: Xue-Mei Lin.

Supervision: Guo-Dong Yang.

Writing – original draft: Cong Yuan.

Writing – review & editing: Guo-Dong Yang, Xue-Mei Lin.

## Footnotes

* The size was not clearly described in the literature.
